# Comparison of tai chi vs. strength training for fall prevention among female cancer survivors: study protocol for the GET FIT trial

**DOI:** 10.1186/1471-2407-12-577

**Published:** 2012-12-05

**Authors:** Kerri M Winters-Stone, Fuzhong Li, Fay Horak, Shiuh-Wen Luoh, Jill A Bennett, Lillian Nail, Nathan Dieckmann

**Affiliations:** 1 School of Nursing, Oregon Health & Science University, Portland, OR, 97239, USA; 2 Knight Cancer Institute, Oregon Health & Science University, Portland, OR, 97239, USA; 3 Oregon Research Institute, Eugene, OR, 97403, USA; 4 School of Medicine, Oregon Health & Science University, Portland, OR, 97239, USA; 5 Portland Veteran’s Affairs Medical Center, Portland, OR, 97239, USA

**Keywords:** Resistance training, Exercise, Physical function, Postural stability, Muscle strength, Chemotherapy, Neoplasm, Fracture

## Abstract

**Background:**

Women with cancer are significantly more likely to fall than women without cancer placing them at higher risk of fall-related fractures, other injuries and disability. Currently, no evidence-based fall prevention strategies exist that specifically target female cancer survivors. The purpose of the GET FIT (Group Exercise Training for Functional Improvement after Treatment) trial is to compare the efficacy of two distinct types of exercise, tai chi versus strength training, to prevent falls in women who have completed treatment for cancer. The specific aims of this study are to: 1) Determine and compare the efficacy of both tai chi training and strength training to reduce falls in older female cancer survivors, 2) Determine the mechanism(s) by which tai chi and strength training each reduces falls and, 3) Determine whether or not the benefits of each intervention last after structured training stops.

**Methods/Design:**

We will conduct a three-group, single-blind, parallel design, randomized controlled trial in women, aged 50–75 years old, who have completed chemotherapy for cancer comparing 1) tai chi 2) strength training and 3) a placebo control group of seated stretching exercise. Women will participate in supervised study programs twice per week for six months and will be followed for an additional six months after formal training stops. The primary outcome in this study is falls, which will be prospectively tracked by monthly self-report. Secondary outcomes are maximal leg strength measured by isokinetic dynamometry, postural stability measured by computerized dynamic posturography and physical function measured by the Physical Performance Battery, all measured at baseline, 3, 6 and 12 months. The sample for this trial (N=429, assuming 25% attrition) will provide adequate statistical power to detect at least a 47% reduction in the fall rate over 1 year by being in either of the 2 exercise groups versus the control group.

**Discussion:**

The GET FIT trial will provide important new knowledge about preventing falls using accessible and implementable exercise interventions for women following chemotherapy for cancer. ClinicalTrials.gov NCT01635413

## Background

Women diagnosed with cancer have a favorable prognosis in terms of survival [1], but will likely face treatment-related side effects and symptoms that threaten quality of life. One of the serious after effects of cancer treatment is an increased risk of falling. Women who have had cancer are significantly more likely to fall than women who have not [2-4] and falls are associated with serious injuries, including fractures [5]. Chen et al. reported an elevated risk of falls after women developed breast or other cancers compared to women never diagnosed with cancer (HR = 1.15, 95%CI: 1.06-1.25 and HR = 1.27, 95%CI: 1.18-1.36 for breast cancer and other cancers, respectively) [6]. In these cancer survivors, hip fracture risk was doubled after diagnosis and multiple fallers (≥2 falls in a year) were significantly more likely to break their hips (HR = 2.05, 95%CI: 1.82, 2.31) or spines (HR = 1.76, 95%CI; 1.58, 1.95) compared to women who fell once or not at all [2]. Since falls increase *after* women are diagnosed with cancer [2], the disease and/or treatments are likely to be the cause of increased falls. Cancer treatment can cause muscle wasting [7-11], peripheral neuropathy in the feet [12] and vestibular ototoxicity [13,14]. and these same problems have been linked to poor balance and falls in older adults [15-17] and persons with diabetes [18-20]. Reduced physical activity, common during and after chemotherapy [19], could further contribute to fall risk because inactivity leads to declines in neuromuscular function.

Although the precise reasons that falls increase after cancer diagnosis remain to be determined, exercise is a fall prevention strategy that has demonstrated efficacy in older adults [20,21] and might also be effective in cancer survivors. In older adults, both strength training and tai chi are types of programs that effectively reduce falls because they each address the underlying reasons people fall in old age, e.g., muscle weakness and poor balance [22-24]. The specific ways that cancer treatment changes fall risk factors suggest that strength training or tai chi might prevent falls in female cancer survivors through different mechanisms. Strength training can prevent falls because strong muscles promote stability that prevents the initiation of a fall [16], and counteracts the downward force of a fall once balance is lost [25]. There is strong evidence that strength training reduces falls by 30%-50% in older adults without cancer [17,24,26-32]. In contrast to strength training, tai chi another standing exercise consisting of a series of individual dance-like movements linked in a continuous sequence, flowing slowly and smoothly from one movement to another that emphasizes weight transfer and movement of the body outside of its base of support [33]. By doing so, it improves postural control and balance [34] that can prevent falls. A recent meta-analysis that pooled the effect of six controlled trials examining the efficacy of exercise to reduce falls in older adults reported a 49% reduction in fall incidence from tai chi (Incidence Rate Ratio (IRR)=0.51, 95% CI: 0.38–0.68) [35]. Falls in BCS have been seldom studied and exercise has only been explored by a singular trial that considered falls as a secondary endpoint [36]. This study failed to find that strength training protected against falls; however, the study did not use a strength training program designed for fall prevention and the study was not powered for falls.

The specific ways that cancer treatment changes fall risk factors suggest that strength training or tai chi may differentially reduce falls in female cancer survivors or may be equally effective in preventing falls. While either strategy reduces falls in older adults, neither has been rigorously tested in cancer survivors. The objective of the GET FIT (Group Exercise Training for Functional Improvement after Treatment) trial is to compare the efficacy of 2 distinct types of exercise, tai chi versus strength training, to prevent falls in women who have completed treatment for cancer. To our knowledge, this study will be the first to test the efficacy of any intervention in cancer survivors with falls as a primary endpoint, to identify the mechanisms through which either intervention reduces falls, and to evaluate whether intervention benefits on falls persist long-term. The primary aim of our study is to determine and compare the efficacy of tai chi training and strength training to reduce falls in older female cancer survivors, with a secondary aim to determine the mechanism(s) by which tai chi and strength training each reduce the risk of falls and, a tertiary aim to determine how well the benefits of each intervention persist long-term. The purpose of this paper is to describe the study protocol for the GET FIT trial and present potential challenges during the study and our approach to dealing with these challenges.

## Methods/Design

### Study design and setting

The study is a single-blind, parallel group, randomized controlled trial comparing 3 groups: 1) tai chi, 2) strength training, and 3) a placebo control. The study period is 12 months including a 6-month supervised intervention and a 6-month follow-up with the primary outcome measured monthly and secondary and process outcomes measured at baseline, 3, 6 and 12 months. The primary site for conduct of the study, including all study visits, is Oregon Health & Science University (OHSU) in Portland, Oregon. Exercise training will be conducted at OHSU and additional community locations serving the outer Portland area. The OHSU IRB has approved the protocol and informed consent for the study. The trial is registered with ClinicalTrials.gov NCT01635413.

### Sample

Participants are underactive women aged 50–75 years who completed chemotherapy for cancers without neurologic involvement. The eligibility criteria are inclusive to demonstrate the feasibility of the intervention in a broad range of female cancer survivors and to enhance the generalizability of the findings. Women with chronic conditions and health problems are eligible unless the problems are serious enough to preclude participation in moderate level exercise. To be eligible to participate in the study women must meet the following inclusion criteria: 1) diagnosed with stage I-IIIc cancer other than cancers of the brain or spinal cord, 2) completed chemotherapy >3 months prior to enrollment and no concurrent adjuvant therapy other than hormone manipulation therapy for breast cancer, 3) aged 50–75 on date of enrollment, 4) currently underactive (<60 minutes of moderate intensity exercise per week in the last month; 5) cognitive ability sufficient to answer survey questions, participate in the exercise classes and performance tests, and provide informed consent and 6) free of any medical condition, movement or neurological disorder, or medication use that contraindicates participation in moderate intensity exercise.

### Power and sample size

The sample size is powered to address the primary study aim to reduce falls. In older adults, 30% of community-dwelling elders are likely to fall within 1 year [37] and Li and others have reported a reduction in fall risk of 47% from 3 [38] or 6 [22] months of tai chi exercise. This effect size provided a realistic estimate of the expected reduction in fall risk and was used as the basis for power analyses. The required sample to test the primary hypotheses in a negative binomial regression model [39] was determined with the following assumptions:, alpha=.05, 25% shared variance (R [2]=.25) among the dummy vectors representing treatment, and an overdispersion parameter (Phi) of 2.5. Under these assumptions a sample size of n=342 (n=114 per group) will provide 80% power to detect at least a 47% reduction in the fall rate over 1 year by being in the 2 experimental groups (tai chi and strength training) versus the control group. These estimates were generated using PASS 2008 [40]. To protect against an estimated attrition of 25% during the intervention period, 143 participants will be randomized per group (total sample size: N=429). The attrition estimate is based on attrition rates in yearlong studies of weighted vest strength training in BCS [41] and slightly higher than what has been observed in 1-year studies of older women without cancer participating in tai chi exercise [42,43] (19%-24%) or resistance exercise (24%) [43].

### Recruitment

Participants will be enrolled into the trial over a 27-month recruitment period, yielding a rate of ~16 participants/month. The planned enrollment period will begin January 2013 and conclude in April 2015. Women will be enrolled in consecutive “waves” of ~n=50 for efficient use of personnel and resources across the study period and to test women close to the start and end of exercise classes. The primary method of recruitment will be through mailings from the Oregon State Cancer Registry (OSCaR), a population-based tumor registry run by the Department of Human services that collects and analyzes information on cancer cases in Oregon. The registry will send an information letter to all living female cancer survivors with eligible diagnoses, between the ages of 50 and 75 years old and within a 30 mile radius of the intervention sites that describes the study and asks them to return a pre-paid response form indicating their willingness to be contacted. Additional recruitment strategies include direct referral through oncology providers at OHSU and other local clinics and direct community recruitment using newspaper ads, radio, announcements on websites, as well as presentations at cancer support groups and cancer-related conferences.

### Procedures

Interested women are screened and those eligible are scheduled for an appointment where they will provide written consent and undergo baseline data collection All data collection is conducted according to standard operating procedures. Participants complete questionnaires first followed by testing for postural stability, physical function, and maximal muscle strength. This testing order is designed to minimize the impact of fatigue on performance measures and is used for the baseline, 3 month, 6 month and 12 month data collection visits. Falls are tracked by each participant on a daily log that is collected by study staff each month.

### Randomization and blinding

After completing baseline testing, each participant will learn her group assignment by receiving a sealed, sequentially numbered envelope from the project director and opening the envelope that contains the group assignment randomly assigned to her sequence number. The sequence numbers are generated by a statistician using MS Excel. Measurement technicians who are responsible for data collection and for coding falls data are blinded to participant group assignment.

### Study interventions

Participants in each study group will attend supervised 1-hr classes, twice weekly for 6 months. It is expected that women with mixed employment status will enroll in the study so daytime and early evening classes will be offered. At each site, a certified exercise instructor trained by the study team will lead classes. Each intervention minimizes reliance on expensive equipment and uses simple, easily performed exercises based on functional movements. It is difficult to precisely equilibrate the total volume (intensity and duration) of exercise performed between experimental groups because the nature of each modality is so different. Both will be matched as closely as possible in progression from the low to high end of the range for moderate intensity. Instructors will work with the study investigators and project director on a monthly basis to refine individual participant efforts based on individual tolerance and to adjust their training progression.

### Strength training

The strength training program used in this study is based on training programs that improved neuromuscular function (strength, gait, and balance) and reduced fall risk factors in our prior studies in women without cancer [44,45] and in our recently completed trials in BCS. This program complies with the American College of Sports Medicine (ACSM) exercise guidelines for cancer survivors [46] and with ACSM recommendations for progressive resistance training for novice weightlifters and older adults for 1–3 sets of 8–10 exercises at a weight that can be done for 8–12 repetitions (approximately 60-80% of 1-rep max) with 1–2 min rest between sets [47]. The strength training intervention will use weighted vests to apply resistance during lower body exercises and steps to increase height during stepping routines*.* Lower body strengthening exercises using functional movement patterns that challenge balance by using muscle groups and movement involved in everyday activities (chair rises, 90-degree squats, side-to-side squats, toe raises, lunges (forward, lateral, backward, walking), multi-directional step ups) will comprise the study program. The intensity of strength exercises, set by the amount of weight in the exercise vest (expressed as a percent of body weight) will increase from 4% of body weight to 15% of body weight and duration for each exercise will range from 1–3 sets of 8–15 repetitions. Training is progressive, where intensity will increase by 2%-3% of body weight per month, sets will increase by ~1 set every 2 months and repetitions will decrease from the high to the low end of the range as intensity and sets increase.

### Tai chi

The Moving for Better Balance program [48] will be the Tai Chi intervention to in this study. The protocol consists of a set of purposeful and functional movement forms blended with practice variation and therapeutic moves developed on the basis of prior studies [22,49-51]. Because the goal of the exercise is to assist patients in retaining postural stability and mobility, the protocol is specifically designed to challenge balance control and train gait patterns, as reflected in movements such as ankle sways, displacement of the body's center of mass over the base of support, trunk rotation/flexion, coordinated eyes-head movements, and multi-lateral stepping. The only resistance applied during exercise will be the participant's own body weight. The early stage of the program (i.e., the first 10–12 weeks) emphasizes primarily learning and practicing single and integrated forms/movements with multiple repetitions. The later stage focuses on performing and exercising integrated forms, variation in forms, and therapeutic moves to improve movement coordination, postural balance, sensory integration, and locomotion. Natural breathing is also emphasized as part of the exercise and integrated into the tai chi movement routine. Training is progressive with the number of forms doubling and repetitions increasing by ~2-4 each month for the first 3 months and the complexity of forms gradually increasing thereafter.

### Stretching control

Participants in the control group will attend a supervised stretching program of the same frequency, duration, and length as the tai chi and strength training groups. During stretching, participants will perform a series of whole body flexibility (stretching) and progressive neuromuscular relaxation exercises. Stretching exercises will be performed according to the ACSM guidelines for flexibility exercise: static stretching that emphasizes major muscle groups with 3–4 repetitions of each stretch held to the point of mild tension but not pain for 10–30 seconds [52]. Stretches will be performed from a seated or lying position in order to minimize weight-bearing forces that might increase muscle strength or postural control. The stretching will also be limited in duration to 15–20 minutes, with the remainder of the class devoted to relaxation exercises that produce no neuromuscular stimulus. Many exercise trials in older adults, including ours and including studies with falls as an outcome, have used a flexibility training control condition and results have shown no effect of this training on outcomes associated with tai chi or strength training [36,53-56]. A recent ACSM Roundtable issuing exercise guidelines for cancer survivors recommended “avoidance of inactivity” among cancer survivors to optimize quality of life [46]. Stretching and relaxation exercise increases range of motion, promotes a sense of wellbeing and may be viewed as a beneficial exercise program for participants randomized to this group. We are including a measure of flexibility in our list of outcomes in order to assess the potential benefit of the stretching program and provide an incentive to participants in this group by including a measure that could improve from stretching exercise.

### Participant safety and side effects

Though injuries associated with any of the study exercise programs have been minimal in prior reports, as with any form of exercise there is a slight risk of injury. The following steps will be taken to reduce the risk of injury and symptoms/side effects that might limit exercise adherence, including: 1) required physician clearance for every enrolled participant, 2) monitoring and early care of musculoskeletal symptoms by the study team that includes an expert in care and prevention of sports-related injuries, and 3) minimizing risk of lymphedema through individually tailored exercise programs, instructor and participant education, and regular monitoring for signs and symptoms. If a participant experiences a cancer recurrence over the course of the study data collected after her recurrence date will be excluded from analysis, but she can continue in the study program with physician clearance.

### Six-month follow-up period

To evaluate the long-term effects of tai chi and strength training on falls and function, women will be followed for an additional 6 months after conclusion of the 6-month supervised intervention. Women will continue to track their falls during the follow-up period using the daily logs submitted each month (see Fall Surveillance below). During the follow-up period, we will obtain information about participants’ exercise using weekly logs. Women will be called at 1 and 3 months during the follow-up period to get verbal reports of their exercise logs and the weekly logs will be collected at the month 12 visit. We will consider participation in community and/or home-based exercise in the analysis for Aim 3 (see Analysis Plan) and will also repeat measures of strength and postural stability in order to better assess both the residual effects of the intervention programs among women who do not exercise in the follow-up period and the influence of continued exercise on strength and balance after formal training stops.

## Measures

### Primary outcome

Falls: In this study, a fall is defined as unintentionally coming to rest on the ground or at some other lower level, not as a result of a major intrinsic event (e.g., stroke or syncope) or overwhelming hazard [57]. Falls will be assessed retrospectively at baseline through 6-month recall to characterize the sample and check for equality of randomization. Falls will be assessed prospectively during the study by monthly reports completed through a web portal or returned by postal and/or electronic mail, methods proven successful in our 2 previous studies and by others, including Dr. Li [3,4,22,58]. In our recently completed observation study of BCS (N=58), we obtained 98% of monthly fall reports over 6 months [3]. If a participant indicates a fall she will then be asked to detail any injury that resulted from each fall. A fall will be considered “injurious” if it results in fractures, head injuries, sprains, bruises, scrapes, or serious joint injuries, or if the participant seeks medical care [57]. Of interest in the study are the number of falls, the number of injurious falls, and medical care resulting from a fall during the intervention period.

### Secondary outcomes

Muscle strength. The 1-repetition maximum (1-RM) for leg press will be used to determine changes in lower extremity muscle strength. The 1-RM test is a safe and effective means of evaluating strength, even in geriatric populations that have never lifted weights before [59,60], in BCS who have strength trained [61-63], and in cancer survivors with lower-extremity lymphedema [64]. We have evaluated maximal strength using the 1-RM technique in 3 studies of BCS (age range: 35–89 yrs) with no adverse events. The 1-RM test will be conducted according to established protocols [52]. We will also consider changes in functional leg strength using the timed chair stand test (see PPB) so that strength changes can also be interpreted in a clinical context [65].

Postural Stability. Computerized dynamic posturography (CDP; Clinical Research System, Neurocom Intl, Inc.) measures postural stability and is used to identify objective patterns of balance problems consistent with neuropathy, vestibular loss, and CNS disorders that contribute to fall risk [66]. The test protocol used in this study is the sensory organization test (SOT), which systematically removes or alters useful information to the patient’s vision or support surface to create sensory conflict situations. These conditions isolate vestibular control of balance, as well as stress adaptive sensory reweighitng by the central nervous system. Equilibrium scores are determined for each condition based on maximum forward-backward postural sway. The weighted average of the scores of all sensory conditions characterizes the overall level of performance as a composite equilibrium score. Sensory ratios are calculated by comparing individual equilibrium scores in specific conditions to identify impairments of individual sensory systems including the vestibular, somatosensory, and visual systems. Equilibrium scores and sensory ratios range from 0–100, where 100 = perfect stability and 0 = an inability to maintain balance and a fall. In this study, we are interested in the composite equilibrium score and also the somatosensory and vestibular sensory ratios, since the latter may show differential effects of the 2 interventions on physiologic systems affected by cancer chemotherapy that impair stability (i.e., neuropathy and vestibular impairment). Test-retest reliability of composite equilibrium scores ranges from 0.72 - 0.93 [67].

Physical Functioning will be measured by the Physical Performance Battery (PPB) [68]. The PPB consists of 3 timed performance tests: 5 repeated chair stands, standing balance, and gait speed over 4 meters. We will consider the time scores of the chair stand test and gait speed separately to examine them as clinical measures of strength and postural stability that might mediate fall risk for Aim 2 (see Analysis Plan). Though not a primary aim of our study, the transformed PPB score can be used to evaluate the effect of the intervention on overall physical function, which would be an important additional benefit of the exercise programs. In older adults, low scores on the PPB are significantly associated with subsequent mobility disability, ADL disability, hospitalization, admission to a nursing home, and mortality [68-71]. The PPB is reliable, sensitive to change [72] and has established norms. Higher scores indicate better physical functioning. Reliability of the PPB is high with intraclass correlation coefficients ranging from 0.88-0.92 for individual tests and the composite score [72].

Flexibility may improve in the control group, so we will measure flexibility in all groups using the standardized Chair Sit and Reach Test for lower body flexibility and Back Scratch Test for upper body flexibility [73]. These tests are designed for older adults with demonstrated validity and reliability in older adults (test-retest 0.95-0.96) [73].

### Descriptive variables

Demographic information, including age, education, income, and others will be measured at baseline by a questionnaire developed for this study. In addition, stage of prior cancer diagnosis, type and dates of cancer treatments, additional adjuvant treatments (i.e., hormone manipulation therapy for breast cancer), presence of other chronic conditions, current medications and health habits will be measured. Clinical measures of weight and height will be made. Updated information will be collected at subsequent visits.

Presence of chronic medical conditions that affect physical functioning will be measured by the Functional Comorbidity Index, which is a self- administered 18-item scale and has stronger associations with SF-36 physical function (R [2]=0.29) than other comorbidity indices [74]. Indivdual item responses from this questionnaire will also be used to inform the exercise instructor about medical conditions that might affect participants’ physical ability to exercise.

Fear of falling may impact a participant’s confidence that she can safely engage in a study exercise program. This factor may also change across the intervention. Though this is not a major outcome in this study, it may provide important information about the population and intervention. The Survey of Activities and Fear of Falling in the Elderly (SAFFE) [75] has 11 items representing activities of daily living associated with fear of falling, mobility, and social activities. The SAFFE score is the average of item responses, with higher scores indicating greater fear of falling and has high internal consistency (intra-class correlation coefficient: 0.91) [75].

Exercise outside the exercise intervention could affect the fall and function outcomes of the study. The CHAMPS Physical Activity Questionnaire for Older Adults, a valid and reliable instrument [67], will be used to measure outside physical activity across the course of the study.

Adherence (% of prescribed sessions completed) is tracked from attendance logs recorded by the exercise instructor. Adherence data is used to describe the dose of exercise received by participants. It is expected that women will occasionally miss classes due to travel, illness, or other reasons. However, it is important to count missed classes to get an accurate estimate of received (exercise) dose and to generalize our findings.

### Data analysis plan

The univariate distribution of each variable will be examined to check for departure from normality and outliers [76]. For each analysis described below, each subject will be analyzed according to the group to which she was randomly assigned and regardless of missing data (i.e., intention to treat). Medical treatment changes and cancer recurrence during the study will be tracked and used to describe the sample and document why participants may not have completed the study. In the unlikely event of a large subgroup, these data could be used for exploratory sub-group analysis to suggest directions for future research. Age, cancer type (breast or other), and time since cancer treatment completion, will be included as covariates in all analyses.

A negative binomial regression model will be used to test the efficacy of tai chi and strength training (versus stretching control) in reducing the total number of falls per participant that occur during the intervention period (from baseline to 6 months). Negative binomial regression is preferred over Poisson regression for modeling the count data because of the overdispersion that is common with actual research data [40,77]. Intervention type will be entered into the model as 2 dummy vectors, with the reference group being the control group. Fall rates for participants who drop out of the study will be adjusted by accounting for their exposure time within the study timeframe. A significant incidence rate ratio (IRR) less than 1.0 for the dummy vector representing tai chi and/or strength training would provide support for the hypothesis that the respective intervention reduced the rate of falls compared to the control group (controlling for covariates).

Muscle strength and postural stability will be tested as potential mediators of the relationship between the exercise interventions and falls. We propose that strength and stability will mediate the effect of each intervention on falls because both constructs will be affected by each intervention and are major risk factors for falls. Though we do expect stronger effects of strength training on strength and of tai chi on stability, how each program specifically reduces falls is uncertain, particularly in women treated for cancer. Mediation analyses will be conducted in a structural equation modeling framework (SEM) implemented in Mplus 7.0. A manifest model will be tested with dummy vectors representing tai chi and strength training as exogenous variables, strength and stability (difference score from baseline to 6 months) as endogenous variables (mediators), and number of falls that occurred from baseline to the end of the intervention as an endogenous variable (outcome). The SEM framework allows for the assessment of indirect (mediating) effects with mixed distributional models, in this case Guassian for both mediators and negative binomial for the outcome. Support for mediation will be determined by assessing the magnitude and significance of the indirect effects (standard errors calculated with the multivariate delta method [78]) from the treatment variables to number of falls through changes in strength and stability. Additional mediation analyses will be conducted replacing composite SOT score as the measure of stability with SOM and VEST scores, and replacing maximal knee extensor force (Nm) and composite SOT with clinical measures of strength (PPB chair stand) and dynamic stability (gait speed).

The persistent effects of the intervention on fall outcomes after formal training stops will be evaluated with a piecewise intent-to-treat negative binomial regression model with the change point occurring at 6 months. Of particular interest is whether the IRRs for the treatment groups differ after the change point. In addition, the moderating effect of participation in an exercise program following the end of the intervention will be examined. Specifically, exercise programs engaged in for least 50% of the follow-up period will be categorized and dummy coded (e.g., infrequent participation versus regular participation). These variables will be entered into a negative binomial regression model along with the product of these dummy vectors and treatment (i.e., interaction effects). Significant interaction effects indicate that continued participation modifies the effect of the intervention on fall rate. With this analytic approach we will be able to determine whether the benefits of the exercise interventions persist with and without continued participation in an exercise program, and whether control participants who begin programs similar to the intervention programs show improvements on the fall rate outcome.

## Discussion

Tai chi and strength training are 2 types of exercise that have been recommended for fall prevention in older adults [21]. The best type of exercise to prevent falls in cancer survivors, however, may or may not be the same as that for older adults and likely depends on which best addresses the underlying causes of falls. The GET FIT trial is the first study to reveal which of 2 targeted exercise programs, each specifically focusing on improving strength or balance, best reduces the risk of falls in women treated for cancer. It is also the first direct comparison of the benefits of tai chi versus strength exercise in any group of cancer survivors. Independent of the intervention, this study will be among the first study to aggressively track falls in female cancer survivors and these data could advance our understanding of the prevalence of falls in the population, the reasons why falls increase after cancer in women, and the consequences of those falls.

Delivery of 3 exercise programs at multiple sites introduces the potential for inconsistent delivery of study programs, so the following steps are taken to assure quality control over intervention delivery: 1) Exercise instructor training sessions delivered by the Intervention Supervisor and Investigators (KWS and FL) includes how to instruct each exercise protocol, training progression, safety considerations, and research conduct specific to the exercise program, 2) Written guidelines provided for each instructor, in addition to hands-on training, that outlines the training protocols and overall study conduct to optimize delivery of the classes in a consistent fashion, 3) Rigorous oversight of class instruction. An Intervention Supervisor is on the study team to consistently oversee proper conduct of exercise classes, participant retention and exercise compliance. S/he will observe classes at all sites on a bi-weekly basis and in conjunction with study investigators (KWS and FL), will work with instructors to minimize instructional differences across sites.

Maximizing participant retention is a primary goal for conduct of the trial. Retention rates in prior exercise studies of similar length by the investigative team and in a similar population are strong (82%-85%).^21, 79^ and similar retention strategies are used for the GET FIT trial and include the following: 1) regular participant phone contact during the study 2) reserved parking place near the OHSU School of Nursing for testing appointments 3) free and close-in parking to exercise classes (as close as 200m for physically limited participants) 4) public transportation costs covered for participants who choose not to drive, 5) $10 remuneration at the end of each measurement appointment as a token of thanks, 6) allocation of every participant to an exercise program, including control group participants, 7) offering exercise classes at various community locations in the study area, and 8) encouragement of group cohesion by promoting social interaction through activities inside and outside of class (i.e., holiday potlucks, coffee/tea dates, team building activities during classes).

## Competing interest

The authors declare that they have no competing interests.

## Authors’ contribution

KWS conceived the study and its design and will coordinate the study and draft manuscripts. FL contributed to the conceptualizing the study and design and will work with KWS to coordinate the study and oversee the tai chi intervention and will contribute to data interpretation and drafting manuscripts. FH contributed to preliminary work that informed conception of the study and will oversee computerized dynamic posturography measures and will contribute to data interpretation and drafting manuscripts. JB contributed to the design and conceptualization of the study and will contribute to oversight of participant retention, interpretation of physical function outcomes and drafting manuscripts. LN will contribute to oversight of participant retention and safety, particularly with respect to lymphedema, interpretation of findings and drafting manuscripts. SWL contributed to preliminary work that informed conceptualization of the study conceptualization will aid in determining eligibility of participants and will contribute to interpretation of study outcomes and drafting manuscripts. ND participated in the design of the statistical approach and will perform the statistical analysis and contribute to manuscript development. All authors read and approved the final manuscript.

## Pre-publication history

The pre-publication history for this paper can be accessed here:

http://www.biomedcentral.com/1471-2407/12/577/prepub
